# Population mental health in Burma after 2021 military coup: online non-probability survey

**DOI:** 10.1192/bjo.2023.550

**Published:** 2023-08-14

**Authors:** Htay-Wah Saw, Victoria Owens, Stephanie A. Morales, Nicolas Rodriguez, Christoph Kern, Ruben L. Bach

**Affiliations:** Michigan Program in Survey and Data Science, University of Michigan, Ann Arbor, USA; Westat, Rockville, Maryland, USA; Michigan Program in Survey and Data Science, University of Michigan, Ann Arbor, USA; University of Mannheim, Mannheim, Germany; Mannheim Centre for European Social Research (MZES), University of Mannheim, Mannheim, Germany

**Keywords:** Burma, military coup, armed conflicts, depression, anxiety

## Abstract

**Background:**

Humanitarian crises and armed conflicts lead to a greater prevalence of poor population mental health. Following the 1 February 2021 military coup in Burma, the country's civilians have faced humanitarian crises that have probably caused rising rates of mental disorders. However, a dearth of data has prevented researchers from assessing the extent of the problem empirically.

**Aims:**

To better understand prevalence of depressive and anxiety disorders among the Burmese adult population after the February 2021 military coup.

**Method:**

We fielded an online non-probability survey of 7720 Burmese adults aged 18 and older during October 2021 and asked mental health and demographic questions. We used the Patient Health Questionnaire-4 to measure probable depression and anxiety in respondents. We also estimated logistic regressions to assess variations in probable depression and anxiety across demographic subgroups and by level of trust in various media sources, including those operated by the Burmese military establishment.

**Results:**

We found consistently high rates of probable anxiety and depression combined (60.71%), probable depression (61%) and probable anxiety (58%) in the sample overall, as well as across demographic subgroups. Respondents who ‘mostly’ or ‘completely’ trusted military-affiliated media sources (about 3% of the sample) were significantly less likely than respondents who did not trust these sources to report symptoms of anxiety and depression (AOR = 0.574; 95% CI 0.370–0.889), depression (AOR = 0.590; 95% CI 0.383–0.908) or anxiety (AOR = 0.609; 95% CI 0.390–0.951).

**Conclusions:**

The widespread symptoms of anxiety and depression we observed demonstrate the need for both continuous surveillance of the current situation and humanitarian interventions to address mental health needs in Burma.

Armed conflicts often have long-standing adverse effects on psychosocial functioning in affected populations, including trauma, poor mental health, long-term health consequences, increased exposure to infectious diseases and an impact on reproductive and maternal health.^1,2^ The impact of violence, armed conflicts and political unrest on population mental health can be immediate, long lasting and substantial.^3–6^ Conflict situations often increase the severity of pre-existing mental disorders and their frequency across the affected populations.^7–11^ Victims internally displaced by conflicts often suffer depressive symptoms, post-traumatic stress disorder (PTSD), anxiety and sleep disturbance.^3,12–14^ Similarly, survivors of conflicts often experience anxiety, depression, stress and insomnia due to loss, lack of safety, displacement, trauma, and food and water shortages.^15^ The lack of psychological support in conflict-ridden areas compounds the effects of conflicts on the mental health of conflict survivors.^15^ The true impact of conflicts on mental health is probably underestimated as the populations most vulnerable to poor mental health in conflict regions are hard to reach.^16^

On 1 February 2021, the Burmese military staged a coup and overthrew a democratically elected civilian government.^17^ Following the coup, anti-coup peaceful protests were organised across the country. Hundreds of thousands of government employees also joined the protests by refusing to continue to work under the military government.^17,18^ The Burmese military responded to the protests with excessive use of force, resulting in violence against anti-coup protesters; violations of human rights; bombardment of residential areas; the arbitrary killing of civilians and children; looting, burning and destruction of private property; and arrests of activists.^17–19^ The crackdown on civilians triggered humanitarian crises across the country; disruptions to the financial industry; closure of hospitals, clinics and schools; limited access to basic healthcare; soaring commodity prices; displacement of civilians; loss of employment and livelihoods; and disruption of communication due to internet cut-offs and travel restrictions.^19–22^ However, owing to a lack of data, there are no studies assessing the population mental health outcome in the post-coup period.

## The current study

We assess the prevalence of symptoms of depression and anxiety among the Burmese adult population following the coup and how they vary across sociodemographic subgroups. Data come from an online survey fielded among 7720 Burmese individuals aged 18 and above in October 2021. Study findings will better inform humanitarian and policy responses to address the population's mental health needs following the coup.

## Method

### Data and study population

We fielded an online survey targeting the Burmese adult population between 7 October 2021 and 14 October 2021 for a total of 8 consecutive days using Facebook for sampling. Participants accessed the survey through Facebook advertisements prepared in the Burmese language, sampling from over 20 million Facebook users in Burma aged 18 and over. The Facebook advertisements appeared at the top of the user's Facebook news feed and linked to our online survey. A total of 1.33 million active Facebook users in Burma aged 18+ saw our advertisments at least once between 7 October 2021 and 14 October 2021. Of these, 31 015 users clicked the link to our online survey and 7720 completed it, resulting in a completion rate of 25%.

A growing number of studies have been relying on social media to recruit survey respondents.^23,24^ For instance, Facebook has been used to collect population data during public health emergencies such as the COVID-19 pandemic.^25,26^ Facebook has been indispensable for accessing the population during political upheavals such as that in Burma, where swift humanitarian responses require timely and actionable data, and in-person interviews are hindered by security, operational, logistical and cost limitations.

We collected information on each respondent's demographics, mental health, employment, trust in financial institutions, COVID-19 status, food insecurity, social safety net, access to healthcare, use of mobile phone services, sources of information used to learn about the crisis, and trust in those information sources, aimed at assessing the socioeconomic consequences of the coup. The online survey took about 16 min on average to complete. The survey questionnaire is shown in the Supplementary material, available at https://doi.org/10.1192/bjo.2023.550. Respondents completed the survey on their tablets, smartphones, laptops and PCs and were compensated MMK 2000 each (about US$1.12).

### Mental health outcome measures

We measured the mental health outcomes using the Patient Health Questionnaire-4 (PHQ-4). This is a valid and reliable measure of anxiety (prevalence of feeling ‘nervous, anxious or on edge’ and not being able to ‘stop or control worrying’) and depression (prevalence of feeling ‘down, depressed or hopeless’ and ‘little interest or pleasure in doing things’) over the previous 14 days^27,28^ and it has good internal consistency (Cronbach's α = 0.82). The response scale for each of the items are 0 = ‘not at all,’ 1 = ‘several days,’ 2 = ‘more than half the days’ and 3 = ‘nearly every day’. The scores for each of the four items are aggregated to analyse the overall mental health outcome, and the two conditions (depression, anxiety) can be summed and scored separately to analyse the severity of each condition. We consider three binary outcomes in this study:

- showing symptoms of both depression and anxiety disorder if scores (range 0–12) on the overall scale are ≥6 (probable depression and anxiety); we include the combined measure of symptoms for anxiety and depression as an indicator of overall mental distress as prior research has done;^29^
- showing symptoms of depressive disorder if the scores (range 0–6) on the depression subscale are ≥3 (probable depression);
- showing symptoms of anxiety disorder if the scores (range 0–6) on the anxiety subscale are ≥3 (probable anxiety).^28^

These cut-off values are indicative of major depressive disorder and generalised anxiety disorder, with high sensitivity and specificity.^30,31^

The probable depression and anxiety measure is based on the absolute score on the overall scale rather than the combination of the individual depression and anxiety subscales. For example, an individual may meet the criteria for both probable anxiety and probable anxiety and depression, yet not meet the criteria for probable depression.

### Demographic variables

We collected demographic information, including gender (male or female); age (18–24 years, 25–30, 31–40, 41–50, 51 and older); ethnicity (ethnic Burmese, non-Burmese ethnic groups); whether participants are married or single; and whether participants’ households have children or not. We also collected socioeconomic information such as educational level (secondary and below, high school diploma, some college, college degree, Master's degree or higher, other); household income (2.5 Lakhs and below, 2.5–4.9 Lakhs, 5 Lakhs and above; the Lakh is a widely used currency unit in Burma; 1 Lakh is equivalent to 100 000 Burmese currency Kyat units or about US$56); and employment status (self-employed, salaried person, farmer/day labourer, unemployed, other).

Instead of income, one could use consumption as a proxy for a household's socioeconomic status. Collection of consumption data is a two-step process: a complete list of durable and non-durable consumption goods (together with their current prices) in the context of the country/population in question is compiled through a pilot study, then the amount or quantity of individual goods consumed in the previous week/month/year by each household is collected in the main survey. A conventional face-to-face household survey is a more appropriate approach to collect consumption data, which normally takes several years to complete. One of this study's aims was to generate actionable data in a timely and cost-effective manner for swift humanitarian responses. Therefore, using household income as a proxy for socioeconomic status offered practical advantages over consumption data.

### Mobile phone subscription

We also collected data on the type of mobile phone services respondents used among the four mobile companies operating in Burma: Mytel, Myanma Posts and Telecommunications (MPT), Ooredoo and Telenor. Mytel is partially owned by the Burmese military, whereas MPT is a state-owned company. Ooredoo and Telenor are private telecommunications companies based in Qatar and Norway respectively. Mytel users are more likely to be affiliated with the military establishment than MPT, Ooredoo and Telenor users, although cell phone subscription is not a perfect measure of affiliation with the Burmese military.

### Use of and trust in sources of information

We asked respondents whether they used specific information sources to learn about the current situation in Burma (response options were ‘Yes’ and ‘No’; respondents could choose multiple sources from the list) and how much they trusted each of them when it came to learning about the crisis in Burma (response options were ‘do not trust at all’, ‘trust somewhat’, ‘trust mostly’, ‘trust completely’). The information sources listed were Myawaddy television (MWD); Myanmar Radio and Television (MRTV); State Administration Council (SAC) spokesperson (SAC is the formal name for the Burmese military regime); social media; internet and online news outlets; local journals/magazines; friends and relatives; foreign media outlets such as Voice of America (VOA) Burmese Section, Radio Free Asia (RFA), BBC, CNN, Channel News Asia (CNA), Aljazeera, or Japanese NHK Broadcaster; and YouTube. We re-coded responses to each of the trust variables, taking a value of 1 for ‘trust mostly’ or ‘trust completely’ and 0 otherwise. MWD is owned and operated by the Burmese military and MRTV is a state-owned broadcaster.

### Translation and back-translation of the questionnaire

The questionnaire was originally prepared in English and was translated into Burmese by two experienced survey consultants. Back-translation into English was performed to ensure full compatibility with the original version.

### Statistical analysis

Following summary statistics for our sample, we report unadjusted sample prevalence rates separately for probable depression and anxiety, probable depression and probable anxiety. We estimate logistic regressions to examine whether the depression and anxiety disorders vary across sociodemographic subgroups based on gender, age, education, ethnicity, marital status, household income, household composition, employment status, cell phone subscription, and political affiliation as proxied by respondents’ use of media sources. For each logistic regression, we report adjusted odds ratios (AORs), 95% confidence intervals (95% CIs) and level of statistical significance. All analyses were conducted using Stata version 17 for Windows.

### Consent and ethics approval

Informed consent was obtained from all participants. The authors assert that all procedures contributing to this work comply with the ethical standards of the relevant national and institutional committees on human experimentation and with the Helsinki Declaration of 1975, as revised in 2008. All procedures involving human subjects/patients were approved by the Institutional Review Board (IRB) at the University of Michigan.

## Results

### Descriptive statistics

Table 1 presents statistics for our sample, including demographic and socioeconomic information. It shows that the majority of respondents mentioned using the state-owned MPT mobile services, followed by the two private companies. Only about 12% subscribed to the military-owned Mytel. Likewise, military-affiliated information sources (MWD, MRTV and SAC) were used by a small fraction of respondents to learn about the current situation in Burma. Trust in the military-affiliated information sources was found to be extremely low. Instead, the vast majority of respondents used other information sources and expressed high levels of trust in, for example, social media, online and offline news outlets, friends and relatives, as well as foreign media outlets.

**Table 1:** Sample demographic composition (*n* = 7720)

Variable	*n* (%)
Gender	
Male	4969 (64.37)
Female	2751 (35.63)
Age, years	
18–24	3214 (41.64)
25–30	1576 (20.42)
31–40	1268 (16.43)
41–50	842 (10.91)
51 and above	819 (10.61)
Educational level	
Secondary and below	1073 (13.90)
High school diploma	2115 (27.40)
Some college but no degree	1469 (19.03)
College degree	2116 (27.41)
Master's and above	635 (8.23)
Others	312 (4.04)
Ethnicity	
Ethnic Burmese	5733 (74.26)
Non-Burmese ethnic groups	1987 (25.74)
Marital status	
Married	2704 (35.03)
Single	5016 (64.97)
Household income	
2.5 Lakhs and below	4336 (56.17)
2.5–4.9 Lakhs	2009 (26.02)
5 Lakhs and above	1375 (17.81)
Children	
Have children	3142 (40.70)
No children	4578 (59.30)
Employment	
Self-employed	1481 (19.18)
Salaried person	1603 (20.76)
Farmer/day labourer	1327 (17.19)
Unemployed	1298 (16.81)
Others	2011 (26.05)
Mobile phone service provider	
MPT	2100 (30.86)
Mytel	811 (11.92)
Ooredoo	1994 (29.30)
Telenor	1901 (27.93)
Source(s) used to learn about current situation	
Myawaddy television (MWD)	304 (1.51)
Myanmar Radio and Television (MRTV)	391 (1.95)
SAC spokesperson	274 (1.36)
Social media	5180 (25.78)
Internet and online news outlets	4721 (23.50)
Local journals/magazines	678 (3.37)
Friends and relatives	2149 (10.70)
Foreign media outlets	4386 (21.83)
YouTube	2008 (9.99)
Trust ‘mostly’ or ‘completely’ in information source	
Myawaddy television (MWD)	193 (2.50)
Myanmar Radio and Television (MRTV)	205 (2.66)
SAC spokesperson	175 (2.27)
Social media	2371 (30.71)
Internet and online news outlets	1987 (25.74)
Local journals/magazines	1157 (14.99)
Friends and relatives	1735 (22.47)
Foreign media outlets	4989 (64.62)
YouTube	1981 (25.66)

MPT, Myanma Posts and Telecommunications; SAC, State Administration Council.

Table 2 presents the prevalence of reporting symptoms of anxiety and depression, depression and anxiety. Overall, our estimated rates are 61% for probable anxiety and depression, 61% for probable depression and 58% for probable anxiety. For comparison, using the same PHQ-4 measures and cut-off points, the prevalence of reporting symptoms for anxiety and depression among US adults aged 18 and above during the COVID-19 pandemic reached their highest levels of 19% and 13% respectively, in April 2020.^32^ With that in mind, our overall results indicate a heightened level of mental distress among the Burmese adult population in the aftermath of the coup.

**Table 2:** Overall prevalence rates of reporting symptoms for anxiety and depression, depression alone and anxiety alone, and ‘fair’ or ‘poor’ overall mental health rating

	Anxiety and depression	Depression	Anxiety	‘Fair’ or ‘poor’ overall mental health
Overall, *n* (%)	4687 (60.71)	4739 (61.39)	4479 (58.02)	5338 (69.15)

### Multivariable analyses

Table 3 presents estimates of multivariable logistic regressions predicting each of the three outcomes. Predictor variables were: (a) gender; (b) age; (c) highest educational attainment; (d) ethnicity; (e) marital status; (f) income; (g) household composition; (h) employment status; (i) use of mobile services; and (j) level of trust in various media sources. We found large and significant differences in mental health outcomes across some subpopulation groups, with the largest differences found between respondents who ‘mostly’ or ‘completely’ trusted MWD and/or SAC and respondents who did not trust them. Respondents who ‘mostly’ or ‘completely’ trusted MWD were significantly less likely than respondents who did not trust MWD to report symptoms of anxiety and depression (AOR = 0.574; 95% CI 0.370–0.889; *P* < 0.05), depression (AOR = 0.590; 95% CI 0.383–0.908; *P* < 0.05) and anxiety (AOR = 0.609; 95% CI 0.390–0.951; *P* < 0.05). We found similar results for respondents who ‘mostly’ or ‘completely’ trusted SAC. Despite showing relatively better mental health, the estimated prevalence rates for probable anxiety and depression, probable depression and probable anxiety were still found to be high among respondents who ‘mostly’ or ‘completely’ trusted MWD (27%, 30% and 25% respectively) or SAC (24%, 26% and 19% respectively).

**Table 3:** Logistic regression estimates for the three outcomes and ‘fair’ or ‘poor’ overall mental health rating

Variable	Adjusted odds ratio (95% CI)			
	Anxiety and depression	Depression	Anxiety	‘Fair’ or ‘poor’ overall mental health
Gender (reference = male)				
Female	1.347***	1.397***	1.284***	1.440***
	(1.209–1.501)	(1.253–1.557)	(1.154–1.428)	(1.282–1.616)
Age group, years (reference = 18–24)				
25–30	1.238***	1.189**	1.319***	1.218**
	(1.063–1.442)	(1.021–1.385)	(1.136–1.532)	(1.036–1.433)
31–40	1.021	0.988	1.067	1.016
	(0.854–1.220)	(0.827–1.181)	(0.895–1.272)	(0.842–1.225)
41–50	0.853	0.843	0.905	0.837
	(0.689–1.057)	(0.681–1.043)	(0.732–1.118)	(0.670–1.045)
51 and above	0.722***	0.732***	0.977	0.936
	(0.573–0.911)	(0.581–0.922)	(0.776–1.231)	(0.733–1.196)
Education (reference = secondary and below)				
High school diploma	0.980	0.924	0.930	1.101
	(0.828–1.160)	(0.781–1.093)	(0.786–1.100)	(0.926–1.308)
Some college but no degree	1.143	1.157	1.078	1.393***
	(0.949–1.376)	(0.960–1.393)	(0.896–1.296)	(1.147–1.691)
College degree	1.220**	1.210**	1.021	1.358***
	(1.014–1.468)	(1.006–1.455)	(0.850–1.225)	(1.121–1.646)
Master's and above	0.779*	0.791*	0.794*	1.165
	(0.606–1.003)	(0.615–1.019)	(0.618–1.021)	(0.889–1.525)
Others	0.922	1.155	0.767*	1.208
	(0.696–1.222)	(0.868–1.537)	(0.580–1.014)	(0.899–1.623)
Ethnicity (reference = ethnic Burmese)				
Non-Burmese ethnic groups	0.937	0.959	0.909*	0.945
	(0.835–1.052)	(0.854–1.076)	(0.811–1.018)	(0.836–1.067)
Marital status (reference = married)				
Single	0.785***	0.868*	0.775***	0.976
	(0.679–0.906)	(0.752–1.001)	(0.673–0.894)	(0.839–1.134)
Income (reference = 2.5 Lakhs and below)				
2.5–4.9 Lakhs	0.836***	0.853**	0.711***	1.000
	(0.739–0.945)	(0.754–0.965)	(0.629–0.802)	(0.877–1.140)
5 Lakhs and above	0.912	0.996	0.872*	0.919
	(0.779–1.068)	(0.850–1.167)	(0.746–1.018)	(0.778–1.085)
Household (reference = have children)				
Have no children	1.025	1.005	1.098*	0.965
	(0.922–1.141)	(0.903–1.118)	(0.988–1.220)	(0.862–1.081)
Employment (reference = self-employed)				
Salaried person	1.175*	1.239**	1.189**	1.332***
	(0.993–1.389)	(1.048–1.466)	(1.007–1.404)	(1.116–1.589)
Farmer/day labourer	1.141	1.204**	1.134	1.111
	(0.959–1.358)	(1.013–1.432)	(0.954–1.347)	(0.930–1.328)
Unemployed	1.686***	1.827***	1.437***	1.980***
	(1.411–2.013)	(1.528–2.184)	(1.208–1.709)	(1.635–2.399)
Others	1.024	1.034	0.966	1.137
	(0.875–1.199)	(0.884–1.210)	(0.826–1.129)	(0.966–1.339)
Mobile phone service provider (reference = MPT)				
Ooredoo	0.998	1.119*	1.045	0.984
	(0.874–1.140)	(0.980–1.279)	(0.917–1.192)	(0.855–1.133)
Telenor	1.027	1.065	1.004	1.048
	(0.899–1.174)	(0.933–1.217)	(0.880–1.144)	(0.909–1.207)
Mytel	0.600***	0.684***	0.680***	0.757***
	(0.504–0.714)	(0.575–0.814)	(0.571–0.809)	(0.632–0.906)
Trust ‘mostly’ or ‘completely’ in information source				
MWD	0.574**	0.590**	0.609**	0.537***
	(0.370–0.889)	(0.383–0.908)	(0.390–0.951)	(0.349–0.825)
MRTV	0.699	0.842	0.559**	0.893
	(0.441–1.108)	(0.534–1.328)	(0.349–0.896)	(0.561–1.420)
SAC spokesperson	0.488***	0.466***	0.443***	0.327***
	(0.296–0.804)	(0.285–0.762)	(0.264–0.743)	(0.201–0.534)
Social media	1.175*	1.159*	1.071	1.120
	(0.999–1.382)	(0.985–1.363)	(0.914–1.255)	(0.943–1.332)
Internet and online news outlets	0.943	0.934	1.045	0.874
	(0.793–1.123)	(0.785–1.111)	(0.881–1.239)	(0.727–1.051)
Local magazines	0.764***	0.826**	0.782***	1.068
	(0.656–0.890)	(0.708–0.962)	(0.673–0.910)	(0.904–1.261)
Friends and relatives	1.000	0.943	1.086	0.939
	(0.872–1.147)	(0.822–1.080)	(0.949–1.243)	(0.812–1.085)
Foreign media outlets	1.957***	1.898***	1.879***	1.900***
	(1.743–2.196)	(1.690–2.132)	(1.676–2.107)	(1.682–2.146)
YouTube	1.072	1.015	1.032	0.948
	(0.936–1.227)	(0.887–1.163)	(0.904–1.179)	(0.820–1.095)
Constant	1.004	0.920	0.978	1.031
	(0.765–1.317)	(0.701–1.206)	(0.747–1.280)	(0.778–1.366)
Observations, *n*	6804	6804	6804	6804
Pseudo *R*^2^	0.056	0.051	0.052	0.055

MPT, Myanma Posts and Telecommunications; MWD, Myawaddy television; MRTV, Myanmar Radio and Television; SAC, State Administration Council.

*P < 0.10, **P < 0.05, ***P < 0.01.

We also found large and significant differences in mental health outcomes by use of mobile services. Military-owned Mytel mobile service users were significantly less likely than users of MPT, Ooredoo and Telenor mobile services to report symptoms of probable anxiety and depression (AOR = 0.600; 95% CI 0.504–0.714; *P* < 0.01), probable depression (AOR = 0.684;95% CI 0.575–0.814; *P* < 0.01) and probable anxiety (AOR = 0.680; 95% CI 0.571–0.809; *P* < 0.01). Nevertheless, among Mytel users, we found high rates of probable anxiety and depression (45%), probable depression (48%) and probable anxiety (44%).

Furthermore, we found small but statistically significant differences in mental health outcomes by sociodemographic characteristics. Females (compared with males), the 25–30 age group (compared with the 18–24 group), respondents with college degrees (compared with those with secondary education and below) and salaried and unemployed individuals (compared with self-employed individuals) had worse mental health outcomes. In contrast, the 51+ age group (compared with the 18–24 group), respondents with Masters’ degrees and above (compared with those with secondary education and below), single persons (compared with married persons) and respondents from high-income households (compared with those from low-income households) were found to have better mental health outcomes. It is worth mentioning that, as most adults in Burma complete middle or high school education, treating ‘No formal education/Primary school education’ as the reference group and ‘Secondary education’ as a separate group (rather than combining these two groups into a reference category, as in the regression analysis) did not alter the study's substantive conclusions (results not shown).

## Discussion

Little is known about the state of mental health in Burma in the aftermath of the coup in 2021. Apart from studies suggesting that similar situations to that in Burma probably cause poor mental health outcomes in an affected population, there was previously no knowledge about the current mental health situation in the country. Our study offers insight into the state of mental health based on results from an online survey, resulting in the first data-set that includes mental health information in post-coup Burma. Our findings show extraordinarily high rates of both probable depression (61.39%) and probable anxiety (58.02%) across the sample. Surprisingly, among almost all population subgroups, the rates for our individual outcome measures were generally consistent with the overall rate, suggesting that the mental health consequences of the coup are substantial and widespread. Our results also suggest that the prevalence rates of depression and anxiety in post-coup Burma are similar to those reported in previous studies during or in the aftermath of armed conflicts.^7^

The worsening mental health found in this study is corroborated by the severity of the economic damage caused by the coup; an assessment by the World Bank suggests that the Burmese economy contracted by 18% in 2021 following the coup and a report by the United Nations Development Programme (UNDP) estimated that nearly half of Burma's population would be living below the national poverty line by early 2022 as a result of the coup.^21,22^ Our data also reveal widespread closure of healthcare facilities and substantial job losses resulting from business shutdowns across the country following the coup, which probably also worsened the mental health of residents of Burma.

The largest differences in mental health outcomes were seen in groups defined by the media sources that respondents trusted. In particular, those who reported trusting the three military-affiliated media outlets (MWD, MRTV, SAC spokesperson) had lower rates of probable depression and probable anxiety. However, less than 3% of the sample belong to this group, overall. In turn, these results suggest that a large majority of the adult population is suffering mentally. Combined with the high rates of probable depression and probable anxiety we observed in general, this speaks to the extent of the problem and the need for interventions and continuous surveillance to improve the well-being of those living in Burma.

### Limitations

#### Data collection method

Although our results suggest high rates of probable depression and anxiety among Burmese adults, this research is limited by the data collection method employed. The sample was a self-selected non-probability sample, which impairs our ability to make inferences from the results. For several reasons, we chose not to create post-stratification weights to compensate for this. First, because the distribution of our outcome measures was quite similar across demographic groups, applying any adjustment would not have a very large impact on the final estimates. Second, the most recent available data is from the 2014 census, so any adjustments we attempted would still be outdated by several years. Finally, attempting this type of adjustment on a non-probability sample will not necessarily yield more accurate estimates.^33^ In addition, because respondents were recruited through Facebook, the sample pool was restricted to residents of Burma on that platform. It is unclear exactly how many Burmese have internet access and use Facebook; an online marketing company using data sourced directly from Facebook via application programming interfaces (APIs) estimated the number of Facebook adult users in January 2021 to be about 26.4 million, just over half of the entire population of Burma,^34^ suggesting that most adults use the platform. However, it is not clear what differences there are between Facebook users and non-users in terms of mental health and its intersections with demographics that might affect the results of our study. It is worth mentioning that the military has full control over the internet in Burma. In the aftermath of the coup, the military selectively cut off internet access to certain regions, such as Sagain, Chin, Kachin and Kayah, where resistance groups held control. Consequently, internet coverage and access tend to be more readily available in military establishments compared with resistance strongholds. However, owing to a lack of baseline and background data, we were unable to assess the extent to which those opposing or supporting the military were underrepresented or overrepresented in our sample.

#### Outcome measure

Limited research exists on the applicability of Western measures of anxiety and depression in Burma. Although the PHQ-4 itself has not been validated in the study population, existing studies describe similar symptoms and perceptions of depression and anxiety in Burma as found in Western cultures.^35^ For example, symptoms of anxiety described by a sample of medics in east Myanmar included faintness, palpitations and shortness of breath.^36^ Hence, there is reason to believe that the high scores obtained reveal actual mental health problems and not merely cultural differences. Nevertheless, although the PHQ-4 is well-validated, the possibility of nuance in presentations of anxiety and depression within or across cultures warrants further research.

#### COVID-19 versus coup effects

The cross-sectional data we used in this study did not allow us to disentangle COVID-19 and coup effects on mental health, as this would require high-frequency (e.g. biweekly or monthly) longitudinal data covering periods before, during and after the coup. However, owing to the sequence of events, we believe that the findings reported in this study mostly reflect the consequences of the coup (which includes the handling of the pandemic by the military government). According to a UNDP Country Report, until August 2020, Burma had among the lowest COVID-19 infection rates in East Asia. A series of stringent containment and mitigation measures put in place since February 2020 by the then democratically elected government managed to control the spread of the virus, as well as earning the government broad public support and praise from the World Health Organization (WHO). In November 2020, Burma held a general election, in which voters overwhelmingly voted for the ruling party. With the government's vaccination programme to be rolled out in early 2021 (coupled with the government's effective management of COVID-19), the International Monetary Fund projected that Burma would return to an average of 6 to 7% annual growth in 2021. The hope of Burma returning to normal economic growth and moving forward with democratic and economic reforms was abruptly ended on 1 February 2021 by the coup, which effectively halted all pandemic responses and recovery plans and precipitated the country's economic collapse.^21,22^

### Future research directions

Understanding the state of mental health in Burma is the first step to improving it. Future research should continue to monitor Burma to understand the trajectory of mental health problems, both overall and in subpopulations. This includes further data collection in general, as well as specifically improving data quality, by, for example, understanding the shortcomings of our data and adjusting for these by comparison with similar situations elsewhere, if such data exist. Alternatively, we could explore new ways of collecting data in the future while prioritising data quality. We have implemented a data collection method that is safe, cost-effective and timely, but at the cost of accuracy. Developing alternative methods of probability-based data collection with these features will yield higher-quality data that will enable more effective response planning. In addition, it is important to understand what kinds of intervention will be most effective and most feasible to mitigate the causes and effects of widespread poor mental health during humanitarian crises such as that in Burma. In conjunction with research on prevalence, this will inform well-planned response strategies. Future studies should also analyse mental health outcomes in other population subgroups that this study did not consider. For instance, Burma saw large population migration from rural to urban areas after the first democratically elected government came to power in 2015. Future studies should include migration (as well as reverse migration) in the regression analyses, given its potential association with mental health outcomes through employment.

### Implications

It is very likely that the current humanitarian crises in Burma will further deteriorate in the coming months as the ongoing violence and armed conflicts show no sign of abating. The international community should monitor the current situation. Given the challenges associated with delivering humanitarian aid directly to the affected population, there is a strong need for the international community to coordinate with local non-governmental organisations (NGOs), faith-based and ethnic organisations to mobilise resources and humanitarian assistance across the country to provide for the mental health needs of the population.

## Supporting information

Saw et al. supplementary material Saw et al. supplementary material

## Data Availability

The data that support the findings of this study are available from the corresponding author, H.-W.S., on reasonable request.
