# Population mental health in Burma after 2021 military coup: online non-probability survey – CORRIGENDUM

**DOI:** 10.1192/bjo.2023.584

**Published:** 2023-11-08

**Authors:** Htay-Wah Saw, Victoria Owens, Stephanie A. Morales, Nicolas Rodriguez, Christoph Kern, Ruben L. Bach

**Keywords:** Burma, military coup, armed conflicts, depression, anxiety

This article was originally published with incorrect affiliations for two authors.

The affiliation of Christoph Kern was originally given as Michigan Program in Survey and Data Science, University of Michigan, Ann Arbor, USA. The correct affiliation is University of Mannheim, Mannheim, Germany

The affiliation of Nicolas Rodriguez was originally given as Department of Statistics, University of Munich (LMU), Munich, Germany. The correct affiliation is Michigan Program in Survey and Data Science, University of Michigan, Ann Arbor, USA.

The errors have been corrected and the online HTML and PDF versions updated.
