# Supplementary material for: Population mental health in Burma after 2021 military coup: online non-probability survey
Source: BJPsych Open. 2023 Aug 14;9(5):e156. doi: 10.1192/bjo.2023.550 (PMC10594092; doi:10.1192/bjo.2023.550)
Supplement: Saw et al. supplementary material [file S2056472423005501sup001.pdf]

Myanmar Coup Impact Assessment Survey

Introduction

Q1. Welcome to the Myanmar Coup Impact Assessment Study. You have been selected to represent millions of people across Myanmar. In this study, you will complete a few questions regarding the current situations in Myanmar. We are interested in assessing how the recent development in Myanmar has affected you, your household, friends, and communities. Your participation is important because it will help the study accurately represent people like you living in Myanmar today. The study benefits from hearing from people of different cultural backgrounds and ethnicities of Myanmar. The survey will take less than 20 minutes to complete.

Your participation is voluntary, and your responses are anonymous and will be treated with strict confidentiality. This study is being conducted by researchers at the University of Michigan. If you have any questions regarding this study, feel free to contact the research team at mmcoupimpactassessment@umich.edu.

As a token of appreciation for your time, we will send you a phone top-up worth MMK 2,000 upon completion of the survey. At the end of the survey, you will be asked to enter you cell phone number and name of your mobile carrier. We will send you phone top-up to the cell phone number you provide within 2 business days upon completion of the survey.

Please print or save a copy of this page for your records.

Please click "Continue" to begin the survey. By clicking “Continue”, I consent to participate in this study.

1. I consent to participate
2. I refuse to participate

Demographics

Q2. First, we'd like to know your age in years. How old are you now? Please enter your age at the box provided below (RANGE: 1-120).

Q4. Which of the following categories best describe your ethnicity? [NOTE: IF YOU CLAIM MORE THAN ONE ETHNICITY, PLEASE SELECT THE ETHNICITY YOU MOST ASSOCIATES YOURSELF WITH]

1. Bamar
2. Kachin
3. Kayar
4. Kayin
5. Chin
6. Mon
7. Rakhine
8. Shan
9. Chinese
10. Indian
11. Others

Q5. What is the highest education you attained?

1. No formal education
2. Primary school (1-4)
3. Secondary school (5-8)
4. High school graduate
5. Technical high school/vocational training schools
6. Diploma
7. Some university, but no degree
8. Bachelor's degree (e.g. BA, BE, BS, etc…)
9. Master's degree (e.g. MSc, MA, MBA, etc…)
10. Professional degree (e.g. LL.B, MBBS, etc…)
11. Doctorate degree (e.g. PhD)
12. Others

Q6. Your gender

1. Male
2. Female

Q7. Your marital status

1. Married
2. Single
3. Divorced
4. Widowed

Q8. Please enter number of people currently living in your household, including you

1. 1
2. 2
3. 3
4. 4
5. 5
6. 6
7. 7
8. 8
9. 9
10. 10 and above

Q9. How many children under 18 are currently living in your household?

1. 0
2. 1
3. 2
4. 3
5. 4
6. 5 and above

Q10. What is your household annual income from all sources of income? Please include income of other household members as well.

1. Less than 2.5 Lakhs
2. 2.5-4.9 Lakhs
3. 5-9.9 Lakhs
4. 10-14.9 Lakhs
5. 15-19.9 Lakhs
6. 20 Lakhs and above

Q11. In which of the following States/Divisions are you currently living?

1. Kachin
2. Kayah
3. Kayin
4. Chin
5. Mon
6. Shan
7. Rakhine
8. Yangon
9. Mandalay
10. Irrawaddy
11. Magwe
12. Sagaing
13. Tanintharyi
14. Bago
15. Naypyitaw

Q12. What is your present religion?

1. Buddhist
2. Christian
3. Muslim
4. Hindu
5. Others

Q13. What is your current occupation
1. Self-employed
2. Civil servant
3. Company employee
4. Day laborer
5. Farmer
6. Housewife
7. Retiree
8. Student
9. Unemployed
10. Others

Mental Health

Q15. Please evaluate your overall mental health at the present time on a 5-point scale. My mental health is:
1. Excellent
2. Very Good
3. Good
4. Fair
5. Poor

Over the past fourteen days, how often have you been bothered by any of the following problems?
Q16. Feeling nervous, anxious, or on edge
Q17. Not being able to stop or control worrying
Q18. Feeling down, depressed, or hopeless
Q19. Little interest or pleasure in doing things
1. Not at all
2. Several days
3. More than half the days
4. Nearly every day

Now think about the past week and the feelings you have experienced. Please tell us if each of the following was true for you much of the time during the past week.

Q20. Much of the time during the past week, did you feel depressed?
1. Yes

2. No

Q21. Much of the time during the past week, did you feel that everything you did was an effort?

1. Yes
2. No

Q22. Much of the time during the past week, was your sleep restless?

1. Yes
2. No

Q23. Much of the time during the past week, were you happy?

1. Yes
2. No

Q24. Much of the time during the past week, did you feel lonely?

1. Yes
2. No

Q25. Much of the time during the past week, did you enjoy life?

1. Yes
2. No

Q26. Much of the time during the past week, did you feel sad?

1. Yes
2. No

Q27. Much of the time during the past week, could you not get going?

1. Yes
2. No

Healthcare

Q29. Is there a public hospital in the town or ward or village where you currently live?

1. Yes
2. No

Display This Question:

If Q29 = 1

Q30. Is the public hospital still open?

1. Yes
2. No

Display This Question:

If Q30 = 1

Q31. Can you still go to the public hospital and get treatment if you get sick?

1. Yes
2. No

Display This Question:

If Q31 = 2

Q32. What are the reasons you can't go to the public hospital and get medical attention? (please select all that apply)

1. The public hospital is facing severe staff shortage
2. The hospital is overwhelmed with patients and faces severe capacity constraint
3. Hospitals are closed
4. Other reasons

Display This Question:

If Q29 = 1

And Q30 = 1

Q33. How likely is it that the public hospital will remain open in the coming 6 months?

1. Very unlikely
2. Somewhat likely
3. Very likely

Display This Question:

If Q29 = 1

And Q30 = 2

Q34. In which of the following year did the public hospital shut down?

1. 2021
2. 2020
3. Before 2020

Display This Question:

If Q34 = 2021

Q35. In which of the following month did the public hospital in your place shut down in 2021?

1. January
2. February
3. March
4. April
5. May

6. June
7. July
8. August

Display This Question:

If Q34 = 2021

Q36. What are the reasons the public hospital in your place shut down in 2021? (please select all that apply)

1. Due to a lack of medical supplies or resources
2. Due to worsening security environment
3. Other reasons

Display This Question:

If Q34 = 2021

Q37. How likely is it that the public hospital will reopen in the coming 6 months?

1. Very unlikely
2. Somewhat likely
3. Very likely

Q38. Is there a private hospital in the town or ward or village where you currently live? (Please do not include General Practitioner Clinics)

1. Yes
2. No

Display This Question:

If Q38 = 1

Q39. Is the private hospital still open?

1. Yes
2. No

Display This Question:

If Q39 = 1

Q40. Can you still go to the private hospital and get treatment if you get sick?

1. Yes
2. No

Display This Question:

If Q39 = 2

Q41. In which of the following year did the private hospital shut down?

1. 2021
2. 2020
3. Before 2020

Q42. Is there a healthcare center operated by a civil society organization in the town or ward or village where you currently live?

1. Yes
2. No

Display This Question:

If Q42 = 1

Q43. Is the healthcare center operated by a civil society organization still open?

1. Yes
2. No

Display This Question:

If Q43 = 1

Q44. Can you get medical treatment at the healthcare center operated by a civil society organization if you get sick?

1. Yes
2. No

Display This Question:

If Q43 = 2

Q45. In which of the following year did the healthcare center operated by a civil society organization shut down?

1. 2021
2. 2020
3. Before 2020

Q46. Is there a general practitioner (GP) clinic in the town or ward or village where you currently live? A GP clinic is operated by a medical doctor with M.B.B.S degree (Bachelor of Medicine and Bachelor of Surgery).

1. Yes
2. No

Display This Question:

If Q46 = 1

Q47. Can you go to the GP clinic and get medical treatment if you get sick?

1. Yes
2. No

COVID-19

Q49. Have you been tested for COVID?

1. Yes
2. No

Display This Question:

If Q49 = 1

Q50. How many times have you been tested?

1. 1
2. 2
3. 3
4. 4
5. 5
6. 6
7. 7
8. 8
9. 9
10. 10 and above

Display This Question:

If Q49 = 1

Q51. How many times have you tested positive?

1. 1
2. 2
3. 3
4. 4
5. 5
6. 6
7. 7
8. 8
9. 9
10. 10 and above
11. None

Display This Question:

If Q49 = 2

Q52. Do you think you have been infected with COVID?

1. Yes
2. No
3. Unsure

Q53. Has a doctor or another healthcare professional diagnosed any member of your family (excluding you) as having or probably having COVID?

1. Yes

2. No

Q54. Do you know anyone in your close friends who have been diagnosed as having COVID?

1. Yes
2. No

Q55. In the past 3 months, did any member of your family die from COVID?

1. Yes
2. No

Q56. In the past 3 months, did anyone in your relatives die from COVID?

1. Yes
2. No

Q57. In the past 3 months, did anyone in your close friends die from COVID?

1. Yes
2. No

Employment

Q58. Are you currently working?

1. Yes
2. No

Display This Question:

If Q58 = 1

Q59. In your primary job, are you working as a salary person or self-employed or a day laborer?

1. A salary person
2. Self-employed
3. A day laborer

Display This Question:

If Q59 = 1

Q60. What is your main occupation?

1. A civil servant
2. An employee at a company
3. An employee at an NGO/INGO

Display This Question:

If Q60 = 2

Q61. Did your company lay off any employees after Feb 1, 2021?

1. Yes
2. No

Display This Question:

If Q61 = 1

Q62. How many employees did your company lay off after Feb 1, 2021?

1. 1-5
2. 6-10
3. 11-20
4. 21-30
5. 31-40
6. 41-50
7. 51 and above

Display This Question:

If Q61 = 1

Q63. Was the layoff a result of the coup?

1. Yes
2. No

Display This Question:

If Q60 = 2

Q64. How likely or unlikely is it that your company will shut down in the coming 12 months?

1. Extremely unlikely
2. Moderately likely
3. Extremely likely

Display This Question:

If Q60 = 3

Q65. Has your organization where you currently work laid off any employees after Feb 1, 2021?

1. Yes
2. No

Display This Question:

If Q65 = 1

Q66. How many employees has your organization where you currently work laid off after Feb 1, 2021?

1. 1-5
2. 6-10

3. 11-20
4. 21-30
5. 31-40
6. 41-50
7. 51 and above

Display This Question:

If Q65 = 1

Q67. Was the layoff a result of the coup?

1. Yes
2. No

Display This Question:

If Q60 = 3

Q68. How likely is it that the organization where you currently work will shut down in the coming 12 months?

1. Extremely unlikely
2. Moderately likely
3. Extremely likely

Display This Question:

If Q59 = 2

Q69. Is your business still operational?

1. Yes
2. No

Display This Question:

If Q69 = 1

Q70. Currently, how many employees does your business employ?

1. Currently, my business doesn’t employ anyone
2. 1- 5
3. 6-10
4. 11-20
5. 21-30
6. 31-40
7. 41-50
8. 51 and above

Display This Question:

If Q69 = 1

Q71. Did you lay off any employee after Feb 1, 2021?

1. Yes

2. No

Display This Question:

If Q71 = 1

Q72. How many staff did you lay off after Feb 1, 2021?

1. 1-5
2. 6-10
3. 11-20
4. 21-30
5. 31-40
6. 41-50
7. 51 and above

Display This Question:

If Q71 = 1

Q73. Was the layoff a result of the coup?

1. Yes
2. No

Display This Question:

If Q69 = 1

Q74. How likely is it that your business will still be operational in the coming 12 months?

1. Extremely unlikely
2. Moderately likely
3. Extremely likely

Display This Question:

If Q69 = 2

Q75. Did you completely or temporarily shut down your business?

1. Complete shutdown
2. Temporary shutdown

Display This Question:

If Q75 = 1

Q76. In which of the following year did you completely shut down your business?

1. 2021
2. 2020
3. 2019
4. 2018

5. Before 2018

Display This Question:

If Q75 = 1

Q77. In which of the following month did you completely shut down your business?

1. Jan
2. Feb
3. March
4. April
5. May
6. June
7. July
8. August
9. Sep
10. Oct
11. Nov
12. Dec

Display This Question:

If Q76 = 2021

And If

Q77 = 2

Or Q77 = 3

Or Q77 = 4

Or Q77 = 5

Or Q77 = 6

Or Q77 = 7

Or Q77 = 8

Or Q77 = 9

Or Q77 = 10

Q78. How many staff did you have to lay off when you completely shut down your business in 2021?

1. 1-5
2. 6-10
3. 11-20
4. 21-30
5. 31-40
6. 41-50

7. 51 and above

Display This Question:

If Q76 = 2021

And If

Q77 = 2

Or Q77 = 3

Or Q77 = 4

Or Q77 = 5

Or Q77 = 6

Or Q77 = 7

Or Q77 = 8

Or Q77 = 9

Or Q77 = 10

Q79. Was the complete shut down in 2021 a result of the coup?

1. Yes
2. No

Display This Question:

If Q75 = 2

Q80. In which of the following year did you temporarily shut down your business?

1. 2021
2. 2020
3. 2019
4. 2018
5. Before 2018

Display This Question:

If Q75 = 2

Q81. In which of the following month did you temporarily shut down your business?

1. Jan
2. Feb
3. March
4. April
5. May
6. June
7. July
8. August
9. September
10. October
11. November

12. December

Display This Question:

If Q80 = 2021

And If

Q81 = 2

Or Q81 = 3

Or Q81 = 4

Or Q81 = 5

Or Q81 = 6

Or Q81 = 7

Or Q81 = 8

Or Q81 = 9

Or Q81 = 10

Q82. How many staff did you have to lay off when you temporarily shut down your business in 2021?

1. 1-5
2. 6-10
3. 11-20
4. 21-30
5. 31-40
6. 41-50
7. 51 and above

Display This Question:

If Q80 = 2021

And If

Q81 = 2

Or Q81 = 3

Or Q81 = 4

Or Q81 = 5

Or Q81 = 6

Or Q81 = 7

Or Q81 = 8

Or Q81 = 9

Or Q81 = 10

Q83. Was the temporary shutdown in 2021 a result of the coup?

1. Yes

2. No

Display This Question:

If Q59 = 3

Q84. Did your employer lay off any employees after Feb 1, 2021?

1. Yes
2. No

Display This Question:

If Q84 = 1

Q85. How many employees did your employer lay off after Feb 1, 2021?

1. 1-5
2. 6-10
3. 11-20
4. 21-30
5. 31-40
6. 41-50
7. 51 and above

Display This Question:

If Q84 = 1

Q86. Was the layoff a result of the coup?

1. Yes
2. No

Display This Question:

If Q59 = 3

Q87. How likely is it that your current employer will shut down the business in the coming 12 months?

1. Extremely unlikely
2. Moderately likely
3. Extremely likely

Display This Question:

If Q58 = 2

Q88. Are you currently looking for a job?

1. Yes
2. No

Display This Question:

If Q88 = 1

Q89. Did you previously work?

1. Yes
2. No

Display This Question:

If Q89 = 1

Q90. In which of the following year did you quit your last job?

1. 2021
2. 2020
3. 2019
4. 2018
5. Before 2018

Display This Question:

If Q89 = 1

Q91. In which of the following month did you quit your last job?

1. January
2. February
3. March
4. April
5. May
6. June
7. July
8. August
9. September
10. October
11. November
12. December

Display This Question:

If Q90 = 2021

And If

Q91 = 2

Or Q91 = 3

Or Q91 = 4

Or Q91 = 5

Or Q91 = 6

Or Q91 = 7

Or Q91 = 8

Or Q91 = 9

Or Q91 = 10

Q92. What was the main reason you quit your last job in 2021?

1. I voluntarily quit my last job
2. My company or organization shut down
3. My employer asked me to leave because the company/organization was losing money
4. Other reasons

Display This Question:

If Q88 = 2

Q93. Please choose one of the following main reason why you are not currently looking for a job

1. I am completely retired from working
2. I am a student
3. I am a housewife
4. I recently closed down my business and decided to retire
5. I am not interested in working
6. I gave up looking for a job because finding a job is difficult
7. Other reasons

Food Insecurity

Q94. In the past 30 days, were you worried you would run out of food because of a lack of money or other resources?

1. Yes
2. No

Q95. In the past 30 days, did you eat less than you thought you should because of a lack of money or other resources?

1. Yes
2. No

Q96. In the past 30 days, did you go without eating for a whole day because of a lack of money or other resources?

1. Yes
2. No

Q97. How is it likely that your household will not be able to purchase enough food in the next six months?

1. Very likely
2. Likely
3. Unlikely
4. Very unlikely

Social Safety New

In the past 30 days, did you or anyone in your household receive cash assistance from each of the following?

Q98. Friends
Q99. Relatives
Q100. Religious organizations
Q101. Social organizations
Q102. NGOs/INGOs
Q103. Government

1. Yes
2. No

In the past 14 days, did you or anyone in your household receive food assistance from each of the following?

Q104. Friends
Q105. Relatives
Q106. Religious organizations
Q107. Social organizations
Q108. NGOs/INGOs
Q109. Government

1. Yes

2. No

Banking

Q110. Do you currently have a bank account?

1. Yes
2. No

Display This Question:

If Q110 = 1

Q111. What is the name of the financial institution with which you currently have account? (If you have accounts with several banks, please report the bank with which you conduct most transactions or you most interact with).

1. KBZ
2. CB
3. MAB
4. AYA
5. AGD
6. UAB
7. MEB
8. Yoma
9. Myanmar Tourism Bank (MTB)
10. Others

Display This Question:

If Q110 = 1

Q112. If you try to withdraw money either at an ATM machine or at a branch office today, do you think you will face restrictions in how much money you can withdraw from banks?

1. Yes
2. No

Display This Question:

If Q110 = 1

Q113. After the coup, have you withdrawn cask from ATM or Bank Branch in person or through an agent most of the time?

1. In-person most of the time
2. Through an agent most of the time

Display This Question:

If Q113 = 2

Q114. On average, how much broker fee in percentage you expect to pay when trying to withdraw money from your bank accounts?

1. 1%
2. 2%
3. 3%
4. 4%
5. 5%
6. 6%
7. 7%
8. 8%
9. 9%
10. More than 10%

Display This Question:

If Q110 = 1

Q115. Have you used Mobile Banking for purchases at Shopping Centers or Electronic Shops because you were not able to withdrew cash from ATM or at Bank?

1. Yes
2. No

Display This Question:

If Q110 = 1

Q116. Since the coup in Feb 1, 2021, have you ever tried to withdraw money from banks because you felt insecure about your bank deposits?

1. Yes
2. No

Display This Question:

If Q116 = 1

Q117. Since the coup in Feb 1, 2021, how much money have you already withdrawn from your bank deposits? (If you have savings at multiple banks, please consider all savings).

1. I have withdrawn less than 10% of money from my bank savings
2. I have withdrawn between 11% and 20% of money from my bank savings
3. I have withdrawn between 21% and 30% of money from my bank savings
4. I have withdrawn between 31% and 40% of money from my bank savings
5. I have withdrawn between 41% and 50% of money from my bank savings
6. I have withdrawn between 51% and 60% of money from my bank savings
7. I have withdrawn between 61% and 70% of money from my bank savings
8. I have withdrawn between 71% and 80% of money from my bank savings
9. I have withdrawn between 81% and 90% of money from my bank savings
10. I have withdrawn more than 90% of money from my bank savings

Display This Question:

If Q110 = 1

Q118. How much do you trust Myanmar banks in protecting your savings?

1. I completely trust
2. I moderately trust
3. I do not trust at all

Display This Question:

If Q118 = 1

Q119. Please tell us the reasons why you completely trust Myanmar Banks.

Display This Question:

If Q118 = 2

Q120. Please tell us the reasons why you moderately rust Myanmar Banks.

Display This Question:

If Q118 = 3

Q121. Please tell us the reasons why you completely rust Myanmar Banks.

SOURCES OF INFORMATION

We are interested in knowing how people in Myanmar find out information about the current situations in Myanmar.

Q123. Which each of the following information sources have you used to learn about the current situations in Myanmar? [RANDOMIZE THE ORDER OF ITEMS IN THE LIST]

1. Myawaddy television (MWD)
2. Myanmar Radio and Television (MRTV)
3. SAC spokesperson
4. Social media such as Facebook, Twitter, Instagram
5. Internet and online news outlets
6. Local journals/magazines
7. Friends and relatives
8. Foreign media outlets such as VOA, RFA, BBC, CNN, CNA, Aljazeera, NHK
9. YouTube
    1. Yes
    2. No

How much do you trust each of the following sources of information when it comes to informing you about the current situations in Myanmar? [RANDOMIZE THE ORDER OF ITEMS IN THE LIST].

Q124. Myawaddy television (MWD)

Q125. Myanmar Radio and Television (MRTV)
Q126. SAC spokesperson
Q127. Social media such as Facebook, Twitter, Instagram
Q128. Internet and online news outlets
Q129. Local journals/magazines
Q130. Friends and relatives
Q131. Foreign media outlets such as VOA, RFA, BBC, CNN, CNA, Aljazeera, NHK
Q132. YouTube

1. Do not trust at all
2. Trust somewhat
3. Trust mostly
4. Trust completely

Digital Security

Q133. How concerned are you about digital threats (e.g., cybercrime, hacking or surveillance)?

1. Extremely concerned
2. Very concerned
3. Concerned
4. Moderately concerned
5. Not concerned at all

Q134. Have you made any digital safety changes since the coup (such as new passwords, login security or new apps)?

1. Yes
2. No
3. Not sure

Q138. If you have any other comments related to the current situation in Burma, please enter in the box provided below.

Phone Refill

Q135. As a token of appreciation for your time, we will send you MMK 2,000 phone top-up. In order for us to do that, we need your phone number. Are you willing to provide us with a valid phone number to which we will send your phone top-up? We assure you that your phone numbers will only be used for transferring your mobile top-up and will not be shared with anyone else.

1. Yes

2. No

Display This Question:

If Q135 = 1

Q136. Please enter your mobile phone number in the box provided below. We will send your phone refill to this number. You will receive your phone refill within 2 days upon completion of the survey.

Display This Question:

If Q135 = 1

Q137. What is your mobile phone carrier for the phone number you provide?

1. MPT
2. Ooredoo
3. Telenor
4. Mytel
